# A pilot study of longitudinal changes in neurocognition, white matter hyperintensities, and cortical thickness in atrial fibrillation patients following catheter ablation vs medical management

**DOI:** 10.1016/j.hroo.2024.01.002

**Published:** 2024-01-10

**Authors:** Hannah Schwennesen, Jeffrey N. Browndyke, Mary Cooter Wright, Marat Fudim, James P. Daubert, Mark F. Newman, Joseph P. Mathew, Jonathan P. Piccini

**Affiliations:** ∗ Duke Heart Center, Duke University Medical Center, Durham, North Carolina; † Division of Geriatric Behavioral Health, Department of Psychiatry and Behavioral Sciences, Duke University Medical Center, Durham, North Carolina; ‡ Division of Cardiovascular and Thoracic Surgery, Department of Surgery, Duke University Medical Center, Durham, North Carolina; § Duke Institute for Brain Sciences, Durham, North Carolina; ‖ Cardiothoracic Anesthesiology Division, Department of Anesthesiology, Duke University Medical Center, Durham, North Carolina; ¶ Duke Clinical Research Institute, Durham, North Carolina; ∗∗ Department of Anesthesiology, University of Kentucky School of Medicine, Lexington, Kentucky

**Keywords:** Atrial fibrillation, Catheter ablation, Magnetic resonance imaging, Cognitive impairment, Dementia

## Abstract

**Background:**

Cerebral microembolization and atrophy complicate atrial fibrillation (AF).

**Objectives:**

We aimed to compare changes in neuroimaging findings between AF patients treated with catheter ablation and those treated with medical therapy.

**Methods:**

In this pilot study, we evaluated differences in the change in regional white matter hyperintensity burden (WMHb) and cognitive function from baseline to 6 weeks and 1 year in patients treated with AF ablation (n = 12) and patients treated with medical management alone (n = 11). Change in cortical thickness over time in Alzheimer’s disease (AD) risk, aging-associated, and shared AD risk/aging regions was also compared between groups.

**Results:**

The mean age was 69.7 ± 5.0 years, 78% of patients were male, 39% had persistent AF, and all received oral anticoagulation. There were no significant differences between groups in the change in cognitive function. At 6 weeks, there were no significant differences in periventricular WMHb changes between groups (0.00 vs 0.04, *P =* .12), but changes in attention/concentration were inversely correlated with periventricular (*P =* .01) and total (*P =* .03) WMHb. Medical management patients demonstrated significantly greater cortical thinning in AD risk regions from baseline to 1 year (*P =* .003).

**Conclusions:**

AF patients who underwent ablation demonstrated less cortical thinning in regions associated with AD risk than patients treated with medical therapy. Larger, prospective studies are needed to better understand the relationship between AF therapies and the development of cognitive decline.

**Key Findings**

▪ Atrial fibrillation patients treated with medical therapy had greater cortical thinning over 1 year than patients treated with catheter ablation.
▪ There was no difference in the change in cognitive function between patients treated with medical therapy and those treated with catheter ablation.
▪ There was no difference in periventricular white matter hyperintensity burden between groups, but changes in attention and concentration were inversely correlated with white matter hyperintensity burden.

## Introduction

Atrial fibrillation (AF) increases the risk of heart failure, stroke, and mortality.^1^ AF impacts neurologic function beyond the sequelae of symptomatic thromboembolic events, as it has been associated with an increased risk of cerebral atrophy and long-term care admission, even after controlling for increased risk of stroke.^2^, ^3^, ^4^ The underlying mechanisms driving this association remain unclear. Prior studies have shown that AF is associated with smaller regional brain volumes^5^ and greater burden of white matter hyperintensities (WMHs), areas of signal hyperintensity in the deep or periventricular white matter that have been associated with greater risk of stroke and cognitive decline.^6^, ^7^, ^8^

Although catheter ablation has been shown to improve maintenance of sinus rhythm when compared with antiarrhythmic drug therapy, its effect on cognitive function in AF patients is unknown.^9^^,^^10^ Prevention of recurrent AF and maintenance of sinus rhythm may preserve cognitive function,^11^^,^^12^ while catheter ablation may be associated with acute silent cerebral embolism and cognitive dysfunction.^13^^,^^14^ To our knowledge, no prior work has examined the differential impact of AF treatment on neuroimaging correlates of cognitive decline.

Therefore, we conducted a pilot study to compare changes in neurocognitive function, abnormal WMH volumes, and regional cortical thickness in AF patients undergoing ablation vs medical management. We hypothesized that catheter ablation would be associated with an acute, transient decline in neurocognitive function but that ablation patients would have less WMH burden (WMHb) and regional cortical thinning than medical therapy patients over the 1-year follow-up period.

## Methods

### Study population

Patients were recruited from the outpatient heart rhythm clinics at Duke University Hospital. Patients had to be 18 years of age and older with electrocardiographic documentation of AF that was symptomatic and required rhythm control in the view of their physicians. We sought to enroll a similar number of patients who were planned to undergo ablation and patients who were to be treated with a pharmacologic rhythm control strategy (medical management [MM]). Patients with asymptomatic AF, those not on oral anticoagulation, those with reversible causes of AF, those with permanent AF, and those with a life expectancy <1 year were excluded. Patients undergoing ablation underwent de novo pulmonary vein isolation with radiofrequency ablation as described previously.^15^

### Definitions and endpoints

The index date was defined as the time of enrollment in the study when patients underwent baseline cognitive testing. The index date occurred prior to catheter ablation for patients in the ablation group. AF recurrence was measured with 12-lead electrocardiograms scheduled at 3, 6, 9, and 12 months following the index date. More frequent monitoring with 12-lead electrocardiograms or ambulatory monitoring was performed among those with symptoms concerning for recurrence. Recurrence was defined as any documented AF lasting ≥30 seconds in the 3 to 12 months following enrollment, as recommended by the most recent consensus statement.^16^

### Cognitive assessment

Neurocognitive testing was performed at baseline (index date), 6 weeks, and 1 year. In accordance with the consensus statement on assessment of neurobehavioral outcomes,^17^ the following tests were included in the assessment battery: (1) the Randt Short Story Memory Test,^18^ (2) the Modified Visual Reproduction Test from the Wechsler Memory Scale,^19^ (3) the Digit Span and Digit Symbol subtests from the Wechsler Adult Intelligence Scale–Revised,^19^ and (4) the Trail Making Test, Parts A and B.^20^ All patients included in the study completed the same assessment battery. All measures were administered in a standardized format by trained study technicians blinded to treatment group and supervised by a licensed clinical neuropsychologist (J.N.B.). Cognitive assessment at each time point occurred just prior to neuroimaging. Assessment battery variables were converted into *z* scores and then grouped into 4 predefined domains (verbal memory, visual memory, attention/concentration, and executive skills). The domain scores were defined as the average of *z* scores from each test in the domain, and a single global cognitive domain variable was calculated by averaging the score across the 4 domains. Change scores were calculated by subtracting the baseline or 6-week score from the follow-up score at either 6 weeks or 1 year; a negative score indicates cognitive decline and a positive score indicates cognitive improvement.

### Neuroimaging data acquisition

All participants underwent standard magnetic resonance imaging (MRI) safety screening at each time point. Any pre-existing bodily implants or materials were screened to ensure that they would be safe for MRI imaging at 3T MRI and within total specific absorption ratio safety limits. Neuroimaging data were acquired on a 3T GE Discovery MR750 MRI scanner (GE Healthcare, Milwaukee, WI) with an 8-channel head coil. Structural neuroimaging sequences included a high-resolution T1-weighted, 3-dimensional fast spoiled gradient-echo scan (oblique axial acquisition, repetition time = 7.036 msec, echo time = 3.02 msec, inversion time = 400 msec, flip angle = 11°, matrix = 256 × 256, 1 mm^3^ isotropic voxels) and a T2 fluid-attenuated inversion recovery scan (FLAIR; oblique axial acquisition, repetition time = 11,000 msec, echo time = 147.24 msec, inversion time = 2250 msec, flip angle 90°, matrix = 128 × 128, 1 × 2 mm^3^ voxels).

### Neuroimaging data processing

The T1-weighted sequence data were segmented into gray, white, and cerebrospinal fluid maps using Statistical Parametric Mapping software (version 12, 7219; Wellcome Institute, London, United Kingdom) for MATLAB (version R2017b, 9.3.0.713579; The MathWorks, Natick, MA) to derive whole brain tissue volumes and allow for measurement of total intracranial volume. WMHs were measured from coregistered T2 FLAIR data that were longitudinally bias-corrected via the MATLAB Lesion Segmentation Toolbox^21^ and subsequently processed using a neural network–guided, automated lesion processing pipeline.^22^ WMHs were assigned to 1 of 3 regional distributions depending on their proximity to the lateral ventricles (within 3 mm, periventricular), cortical gray/white matter junction (within 3 mm, juxtacortical), or >3 mm from ventricles or gray/white matter junction (ie, deep white matter). Differences in these 3 regional WMH distributions and their totals were examined between groups and over time using a WMHb index reflecting the percentage of hyperintensity volume (in mL) within periventricular, juxtacortical, or deep white matter regions relative to total white matter volume. The WMHb variables and their change over time were subsequently compared with the cognitive assessment outcomes.

Surface-based morphometry cortical thickness measurements at baseline, 6 weeks, and 1 year were derived from the T1-weighted imaging sequence data preprocessed using the Computational Anatomy Toolbox (CAT12) (version 12.6, r1450; University of Jena, Jena, Germany) for MATLAB. CAT12 uses a projection-based thickness method^23^ for surface-based morphometry with topology correction, spherical inflation, and registration for intersubject analysis that mitigates partial volume, sulcal blurring, and asymmetry effects.^24^ Additionally, CAT12 has an integrated longitudinal segmentation pipeline that uses inverse-consistent image realignment and intrasubject bias correction optimized for the detection of subtle volumetric effects over time. Participants’ baseline, 6-week, and 1-year data were used to derive a mean image of all time points, which was then used for spatial normalization estimation. Tissue assignment was estimated using a standardized ICBM tissue probabilistic atlas, affine regularized using the ICBM European brain space template, and normalized to Montreal Neurological Institute atlas space. WMHs were set as their own tissue class during segmentation. CAT12 medium inhomogeneity (bias) correction, medium noise correction, medium local adaptive segmentation, and high processing accuracy settings were used. A 0.5-mm voxel size was selected for internal isotropic cortical thickness estimation, while a scale intensity value of 0.7 was used for initial gray/white matter border surface creation. Participants’ cortical thickness maps for each follow-up time point were subtracted from their baseline to create cortical thickness change maps used for groupwise analyses of change differences.

To explore the regional cortical thickness differences in regions associated with age-related neuropathological decline (Alzheimer’s disease [AD]) and normal aging, we examined our surface-based morphometry data using previously published AD risk, normal aging, and shared AD risk/normal aging regions of interest (ROIs).^25^^,^^26^ Left and right hemispheric and total cortical mean cortical thickness values were extracted from HCP-MMP1.0 atlas^27^ ROIs that encompassed 5 AD risk regions (medial temporal/entorhinal cortex, inferior temporal, temporal pole, superior parietal, and precuneus/posterior cingulate cortex), 7 aging-associated regions (calcarine, caudal insula, cuneus, caudal fusiform, dorsomedial frontal, lateral occipital, precentral), and 4 AD risk and aging shared regions (angular gyrus, supramarginal gyrus, superior frontal, middle frontal).

### Statistical analysis

Baseline patient demographic and clinical factors were summarized by mean (SD) or median (interquartile range [IQR]) for numeric data and number and percentage for categorical data. Differences between groups at baseline were assessed via standardized differences. Cognitive battery domain and global cognitive scores were summarized via mean ± SD and were compared between treatment groups via *t* tests. Differences between the groups in total WMHb were summarized via median (IQR) and compared with Hodges-Lehmann location shift estimation^28^ and Wilcoxon rank sum tests. The associations between total and regional WMHb and cognitive change were assessed via Spearman correlation.

Mean baseline cortical thickness maps, as well as mean cortical thickness change maps at each follow-up time point, were generated for visualization of intragroup longitudinal change. Extracted mean cortical thickness change values (ie, 6 weeks – baseline; 1 year – baseline) for the 3 ROI groupings were mean averaged within hemisphere and then for hemispheres combined and were subjected to Mann-Whitney *U* analysis between groups. The associations between cortical thinning and cognitive change were assessed via Spearman correlation in SAS version 9.2 statistical software (SAS Institute, Cary, NC).

### Study approval

The study was approved by the Duke University Institutional Review Board. All participants provided informed consent prior to inclusion in the study. The research reported in this article adhered to the Helsinki Declaration guidelines.

## Results

### Baseline characteristics and AF in follow-up

Overall, there were 23 patients enrolled in the study, including 12 patients who underwent catheter ablation and 11 patients who were managed medically with antiarrhythmic drug therapy (MM group). As shown in Table 1, the cohort had a mean age of 69.7 ± 5.0 years, 78.3% were male, and 39.1% had persistent AF. Patient characteristics were similar in the ablation and MM groups. Notably, the left atrial diameters (4.4 ± 1.0 cm vs 4.4 ± 1.2 cm), CHA_2_DS_2_-VASc (congestive heart failure, hypertension, age ≥75 years, diabetes mellitus, prior stroke or transient ischemic attack or thromboembolism, vascular disease, age 65–74 years, sex category) scores (2.6 ± 1.3 vs 2.3 ± 0.7), and frequency of persistent AF (n = 4 of 12 vs n = 5 of 11) were similar between groups. Recurrent AF within 12 months was much more frequent in MM patients (n = 12 of 12) than in ablation patients (n = 1 of 12); 100% of ablation patients were in sinus rhythm at 12-month follow-up compared with 45% of MM patients. Two patients in the MM group underwent ablation at 3 months. Among all patients who underwent catheter ablation, the rate of major complications (including ischemic stroke, access site bleeding, acute heart failure, and pericardial effusion) was 0% (n = 0 of 12).

**Table 1:** Baseline and follow-up characteristics by treatment strategy

	Ablation (n = 12)	Medical management (n = 11)	Standardized difference∗
Demographics			
Age, y	69.1 ± 5.5	70.4 ± 4.5	0.255
Male	10 (83.3)	8 (72.7)	0.258
Years of education	17.0 (14.0–19.0)	17.0 (15.0–19.0)	0.244
Diabetes	2 (16.7)	2 (18.2)	0.040
Prior myocardial infarction	2 (16.7)	0 (0.0)	0.633
History of CABG	1 (8.3)	0 (0.0)	0.426
LVEF, %	55.0 (50.0–60.0)	52.5 (47.5–57.5)	0.026
Hypertension	10 (83.3)	10 (90.9)	0.228
Prior stroke or TIA	1 (8.3)	3 (27.3)	0.511
Left atrial diameter, cm	4.4 ± 1.0	4.4 ± 1.2	0.009
Persistent AF	5 (42)	5 (45)	0.076
CHA_2_DS_2_-VASc	2.6 ± 1.3	2.3 ± 0.7	0.300
Time from AF diagnosis to enrollment, d	384 (198–1436)	1971 (976–2998)	0.947
AF follow-up characteristics			
In AF at 12 mo	0 (0)	6 (55)	1.549
Antiarrhythmic drug therapy at 12 mo	3 (25)	4 (36)	0.248
AV nodal blocking agent at 12 mo	10 (83.3)	10 (90.9)	0.218
Anticoagulation at 12 mo	11 (91.7)	10 (90.9)	0.280
Heart rate at 12 mo, beats/min	59.6 ± 13.6	74.5 ± 13.1	1.123
Any recurrent AF over 12 mo†	1 (8)	10 (91)	2.928

Values are mean ± SD, n (%), or median (interquartile range).

AF = atrial fibrillation; AV = atrioventricular; CABG = coronary artery bypass grafting; CHA_2_DS_2_-VASc = congestive heart failure, hypertension, age ≥75 years, diabetes mellitus, prior stroke or transient ischemic attack or thromboembolism, vascular disease, age 65–74 years, sex category; LVEF = left ventricular ejection fraction; TIA = transient ischemic attack.

∗ Standardized difference based on Cohen’s *d* (values <0.1 are negligible, values 0.2–0.8 are moderate differences, and values >0.8 are large differences).

† Excluding AF 0–3 months postablation.

### Change in white matter disease

At baseline, total WMHb was not statistically different between the treatment groups (*P =* .28) (Table 2). There was a significant increase in total (0.17 [IQR 0.02–0.27]; *P <* .01), periventricular (0.09 [IQR 0.02–0.27], *P <* .01), and juxtacortical (6.89 × 10^–4^ [IQR 0.00 to 1.68 × 10^3^], *P =* .05) WMHb from baseline to 1 year in the overall cohort. There was a possible trend toward greater increase in periventricular lesion burden in the MM group at 6 weeks (0.00 vs 0.04, *P =* .12) but not at 1 year (0.08 vs 0.19, *P =* .25).

**Table 2:** Summary of white matter lesion burden at baseline and change over time according to treatment strategy

	Overall	Ablation	Medical management	Hodges-Lehmann difference∗	Wilcoxon *P* value
Baseline lesion burden†					
Total lesion burden, %	0.38 (0.20 to 0.90)	0.61 (0.27 to 1.27)	0.22 (0.16 to 0.51)	0.181	.277
Periventricular lesion burden, %	0.34 (0.19 to 0.84)	0.57 (0.25 to 1.21)	0.21 (0.15 to 0.47)	0.224	.156
Juxtacortical lesion burden, %	0.00 (0.00 to 0.00)	0.00 (0.00 to 0.00)	0.00 (0.00 to 0.00)	0.000	.714
Deep white lesion burden, %	0.02 (0.00 to 0.04)	0.03 (0.00 to 0.05)	0.01 (0.00 to 0.02)	0.017	.235
Baseline to 6-wk change‡					
Total lesion burden, %	0.02 (–0.09 to 0.12)	0.00 (–0.21 to 0.09)	0.03 (0.01 to 0.12)	0.056	.307
Periventricular lesion burden, %	0.01 (–0.09 to 0.11)	0.00 (–0.26 to 0.05)	0.04 (0.01 to 0.13)	0.098	.121
Juxtacortical lesion burden, %	0.00 (0.00 to 0.00)	0.00 (0.00 to 0.00)	0.00 (0.00 to 0.00)	0.000	.974
Deep white lesion burden, %	0.00 (–0.01 to 0.01)	0.00 (–0.01 to 0.01)	0.00 (–0.01 to 0.01)	0.001	.843
Baseline to 1-y change‡					
Total lesion burden, %	0.17 (0.02 to 0.27)	0.08 (–0.01 to 0.23)	0.22 (0.08 to 0.35)	0.086	.275
Periventricular lesion burden, %	0.09 (0.02 to 0.27)	0.08 (–0.01 to 0.23)	0.19 (0.05 to 0.30)	0.098	.245
Juxtacortical lesion burden, %	0.00 (0.00 to 0.00)	0.00 (0.00 to 0.00)	0.00 (0.00 to 0.00)	0.000	.972
Deep white lesion burden, %	0.00 (0.00 to 0.00)	0.00 (0.00 to 0.00)	0.00 (–0.01 to 0.00)	0.008	.159
6-wk to 1-y change‡					
Total lesion burden, %	0.10 (0.04 to 0.20)	0.07 (0.00 to 0.26)	0.11 (0.04 to 0.20)	0.023	.597
Periventricular lesion burden, %	0.08 (0.01 to 0.16)	0.12 (–0.01 to 0.16)	0.07 (0.01 to 0.22)	0.017	.805
Juxtacortical lesion burden, %	0.00 (0.00 to 0.00)	0.00 (0.00 to 0.00)	0.00 (0.00 to 0.00)	0.001	.275
Deep white lesion burden, %	0.00 (–0.01 to 0.01)	0.00 (–0.02 to 0.08)	0.00 (–0.01 to 0.00)	0.008	.438

Values are median (interquartile range).

∗ Hodges-Lehmann Difference is the median of all pairwise differences between the two groups and corresponds to the Wilcoxon linear rank statistic.

† Lesion burden is the percent of white matter assumed by white matter hyperintensities

‡ There are 12 ablation and 10 medical management patients with baseline and 6-week volumetric data. There are 11 ablation and 10 medical management patients with 1-year volumetric data.

### Change in cortical thickness in ROIs

**Figure 1:**
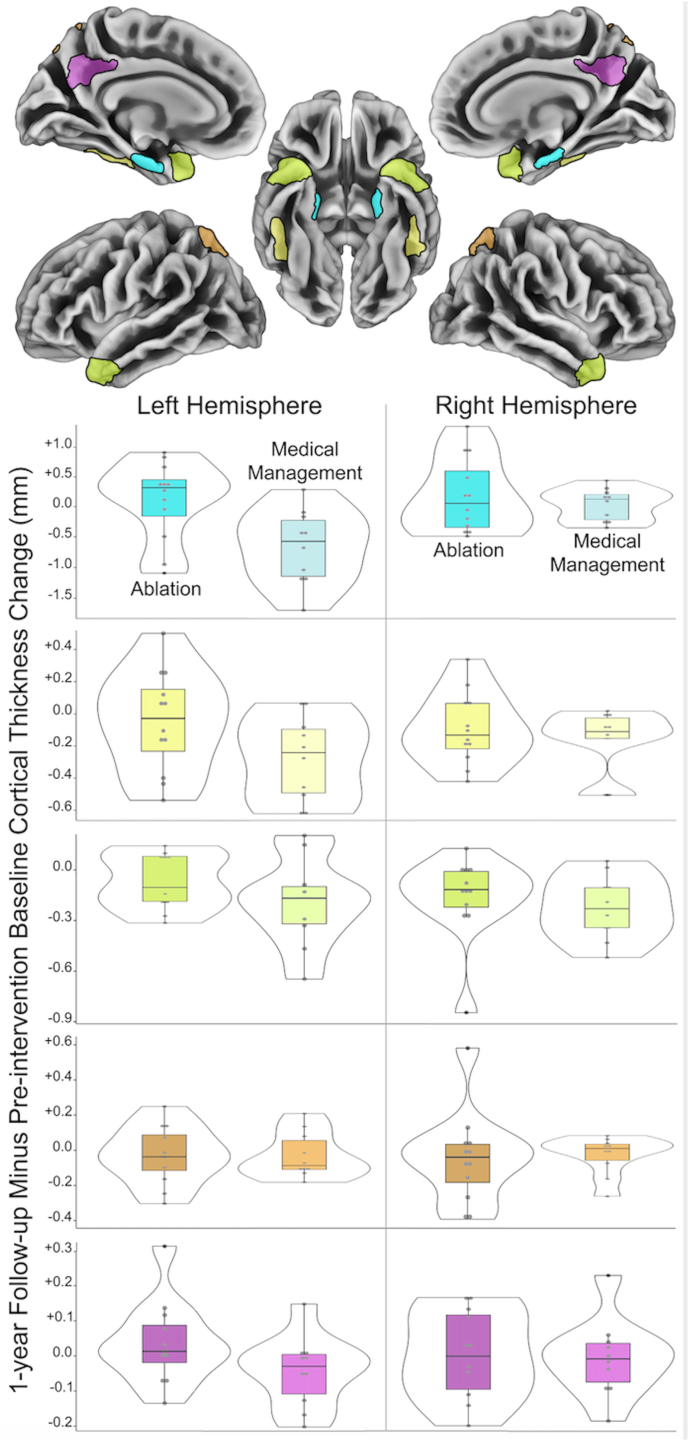
Mean cortical thickness value changes over 1 year in Alzheimer’s disease risk regions. Blue regions of interest (ROIs) = left and right entorhinal cortices; yellow ROIs = left and right inferior temporal gyri; green ROIs = left and right anterior temporal lobes; orange ROIs = superior division of lateral occipital cortices; purple ROIs = posterior cingulate/precuneus cortices. Violin plots show frequency distribution of mean baseline to 1-year cortical thickness change in each group, while internal bars represent the interquartile range and the median change.

There were no statistically significant differences in the change in lateralized or combined hemisphere cortical thickness values in AD risk, aging-associated, or shared AD risk/aging ROI groupings between AF ablation and MM groups at baseline or from baseline to 6 weeks. There was significantly greater left hemisphere and combined hemisphere cortical thinning in the MM group compared with the ablation group from baseline to 1 year in AD risk ROIs (left hemisphere: ablation 0.0003 ± 0.1498, MM –0.0199 ± 0.1436, *P =* .003; combined hemispheres: ablation –0.0098 ± 0.1439, MM –0.1589 ± 0.1346, *P =* .002), driven largely by changes in the left entorhinal cortex (Figure 1). An additional statistically significant change in cortical thickness from baseline to 1 year was revealed for left hemisphere aging ROIs (ablation –0.0131 ± 0.046, MM –0.0222 ± 0.0684, Mann-Whitney *U z* score = 2.2089, *P =* .027), while no statistically significant differences were observed between groups in combined AD risk/aging ROIs from baseline to 1 year.

### Relationship between neuroimaging findings and cognition

**Figure 2:**
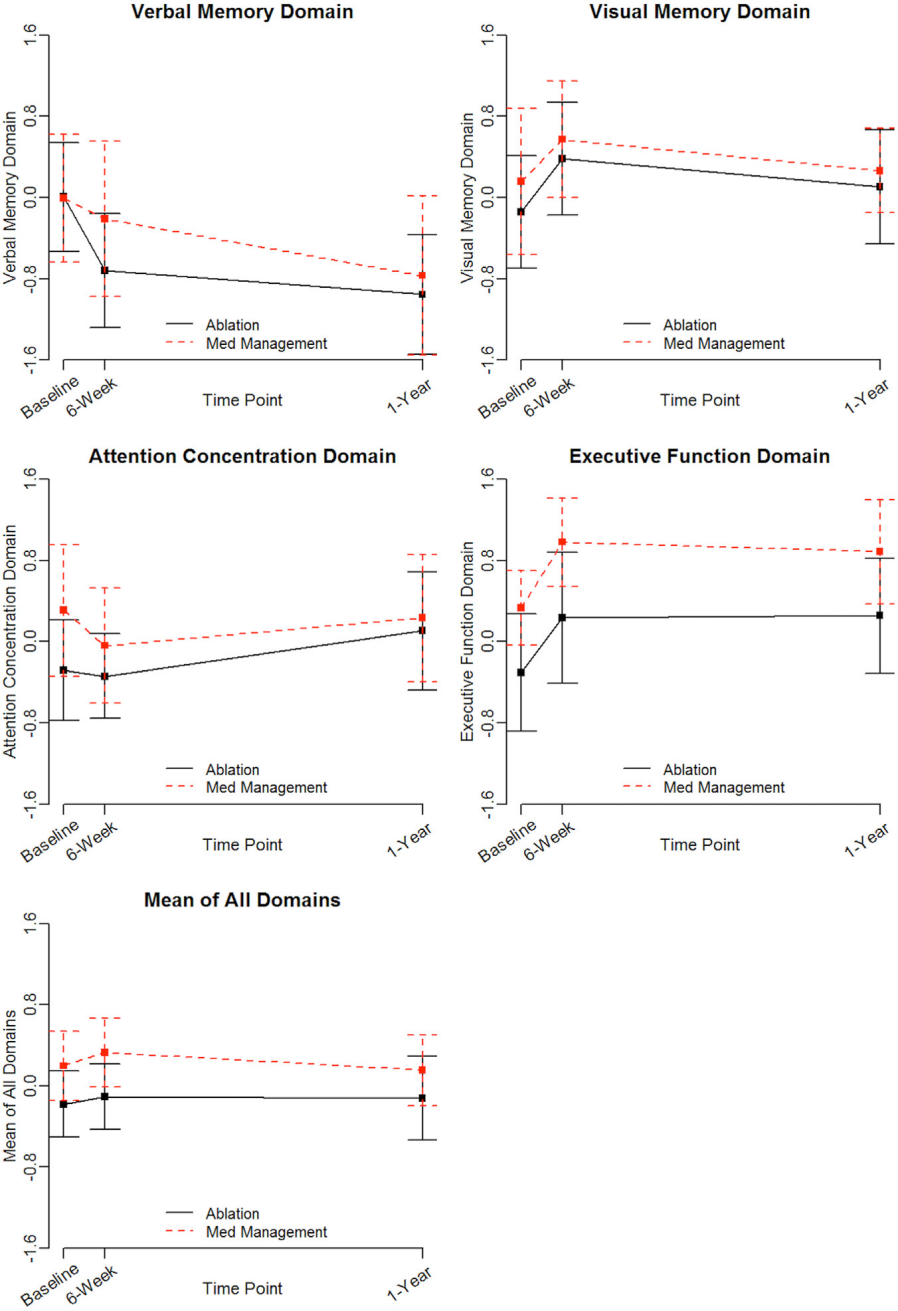
Cognitive trajectories by treatment strategy.

There were small differences in cognitive function between groups at baseline; MM patients had superior performance on executive function tests (0.33 ± 0.55 vs –0.30 ± 0.91, *P =* .05, standardized difference = 0.85) and higher scores on the mean of all domains (0.20 ± 0.51 vs –0.18 ± 0.51, *P =* .09, standardized difference = 0.74). There were no significant differences between groups in any of the 4 cognitive domain scores or the mean of all domains from baseline to 6 weeks or 1 year (Figure 2).

There were several significant findings when assessing the relationship between changes in cognitive function and neuroimaging findings. In the overall cohort, change in attention/concentration from baseline to 6 weeks was inversely correlated with total (r = –0.47, *P =* .03) and periventricular (r = –0.51, *P =* .01) WMHb. Change in global cognitive score from 6 weeks to 1 year was also inversely correlated with total (r = –0.46, *P* = .04) and periventricular (r = –0.47, *P* = 0.03) WMHb. There were no differences between the MM and ablation groups in the relationship between change in cognitive scores and total or periventricular WMHb. Change in cortical thickness in AD risk ROIs was positively correlated with baseline to 1-year change in verbal memory in the ablation group (ρ = 0.60, *P =* .043) but not in the MM group (ρ = 0.15, *P =* .682).

## Discussion

There are several notable findings in this pilot study of neurocognition, WMHs, and cortical thinning in AD risk ROIs in patients with AF undergoing ablation vs medical management. Patients in the MM group demonstrated significantly greater cortical thinning in AD risk regions over 1 year compared with patients in the ablation group. There was no significant difference between groups in the change in periventricular WMHb over time. Although there was no significant difference in cognitive function change between the groups, there was an inverse association between change in cognitive function and WMHb at several time points.

Numerous studies have demonstrated an association between AF and cognitive decline and dementia.^4^^,^^29^ A longitudinal analysis of ∼5000 patients without baseline AF or stroke history showed that incident AF patients were more likely to develop cognitive impairment or dementia at younger ages than those without AF, even after controlling for incident stroke.^29^ A large study of ∼37,000 individuals over 5 years showed that AF was independently associated with the development of all dementia types (including vascular, senile, Alzheimer, and nonspecified).^30^ Despite numerous studies demonstrating the efficacy of rate and rhythm control strategies in reducing AF-related symptoms and stroke risk, their effect on cognition is incompletely understood. Ablation for AF is associated with a small but measurable risk of periprocedural thromboembolism^13^ and has been associated with a 13% to 20% prevalence of cognitive decline at 3 months.^14^ More recently, however, a study of 308 patients demonstrated that catheter ablation of AF does not result in cognitive decline in the short term and that restoration of sinus rhythm might actually improve cognitive performance at 1 year.^11^

WMHs are patchy areas of hyperintense signals seen on MRI that result from demyelination and axonal loss as well as blood-brain barrier dysfunction and serve as markers of cognitive decline and AD.^6^^,^^31^, ^32^, ^33^ Patients with AF demonstrate greater regional WMHs.^7^^,^^8^ Mayasi and colleagues^8^ found that periventricular and confluent deep WMH were significantly greater in AF patients relative to non-AF stroke control subjects, leading to a postulated link between AF and WMH via chronic microembolization. We found that in both the ablation and MM groups, WMHb increased significantly from baseline to 1 year, suggesting that patients treated with catheter ablation and antiarrhythmic drug therapy are both impacted by systemic factors that perpetuate the accumulation of chronic microvascular white matter disease. Our pilot data did not demonstrate any difference in the change in cognitive function between the MM and ablation groups, perhaps due to the pilot study’s small sample size and short follow-up interval. The lack of between-group difference in the change in cognitive function from baseline to 6 weeks suggests that catheter ablation is not associated with acute cognitive decline secondary to periprocedural embolic events, in line with conclusions drawn from other studies that have investigated cognitive function after ablation.^11^^,^^34^ Changes in attention/concentration were strongly correlated with total and periventricular WMHb in the entire cohort, which is consistent with other literature on cognitive decline and WMH.^35^

Because AF has been previously associated with brain atrophy,^2^^,^^36^ we examined differences in cortical thickness change in AD risk regions. From baseline to 1 year, there was significantly greater cortical thinning in MM patients than in ablation patients in AD risk regions.^25^^,^^26^ The greatest difference was seen in the left entorhinal cortex, an area of the brain that plays an important role in memory and navigation through its relationship with the limbic system.^37^ Furthermore, there was a significant positive correlation between cortical thinning in AD risk regions and verbal memory, particularly in the left entorhinal region. These findings raise the hypothesis that catheter ablation may mitigate brain atrophy that eventually contributes to the increased risk of dementia with long-standing AF. One potential mechanism of this effect may be a reduction in AF burden with catheter ablation, as prior work has demonstrated that permanent or persistent AF is more strongly associated with global brain atrophy than paroxysmal AF.^2^

There are several limitations that should be considered when interpreting these results. First, these data and analyses are derived from an observational pilot study with a small sample size; all findings are considered hypothesis-generating and should be tested in larger prospective cohort studies, which would allow for more detailed examination of the association between neuroimaging findings and cognitive outcomes. The study population reflects a relatively healthy AF population, as most patients included in the study had low CHA_2_DS_2_-VASc scores and preserved ejection fraction; therefore, these findings are not relevant for sicker populations of AF patients. Notably, median time from diagnosis of AF to enrollment was significantly longer in patients in the medical management group compared with patients in the ablation group. Greater lifetime exposure to AF may have impacted response to therapy over the follow-up period, leading to differences in neurologic changes between groups. While our study benefits from baseline, postprocedure, and long-term follow-up imaging, imaging was not performed immediately after ablation; therefore, our ability to document acute procedure-associated changes was limited. Future studies should capture more acute postprocedural lesions to discriminate WMH and volume changes associated with embolic events from those caused by chronic AF-related systemic effects. Finally, all patients were anticoagulated according to guideline recommendations, and all ablation patients were heparinized before transseptal puncture and underwent radiofrequency ablation. Therefore, our results may not be generalizable to patients treated with different ablation techniques or anticoagulation protocols.

## Conclusion

Patients treated with medical therapy demonstrated greater cortical thinning in established AD risk regions over time than patients treated with catheter ablation. There were no differences in the change in cognitive function between groups, but there was an association between change in WMHb and deficits in attention/concentration. The potential associations between WMHb, cognition, and progression and dementia should be tested in larger prospective studies with appreciation for both microembolic effects and chronic white matter lesion processes.
